# Cross-sectional study on relationships between physical function and psychological readiness to return to sport after anterior cruciate ligament reconstruction

**DOI:** 10.1186/s13102-022-00491-5

**Published:** 2022-06-01

**Authors:** Junya Aizawa, Kenji Hirohata, Shunsuke Ohji, Takehiro Ohmi, Sho Mitomo, Hideyuki Koga, Kazuyoshi Yagishita

**Affiliations:** 1 Department of Physical Therapy, Faculty of Health Science, Juntendo University, 3-2-12 Hongo, Bunkyo-ku, Tokyo, 113-0033 Japan; 2 Department of Rehabilitation Medicine, Graduate School of Medical and Dental Sciences, Tokyo Medical and Dental University, Tokyo, Japan; 3 Clinical Center for Sports Medicine and Sports Dentistry, Tokyo Medical and Dental University, Tokyo, Japan; 4 Department of Joint Surgery and Sports Medicine, Graduate School of Medical and Dental Sciences, Tokyo Medical and Dental University, Tokyo, Japan

**Keywords:** ACL-RSI, Knee strength, Leg anterior reach, Single-leg hop

## Abstract

**Background:**

Information about specific factors of physical function that contribute to psychological readiness is needed to plan rehabilitation for a return to sports. The purpose of this study was to identify specific physical functions related to the psychological readiness of patients aiming to return to sports 6 months after reconstruction. We hypothesized that the knee strength is a factor related to the Anterior Cruciate Ligament–Return to Sport after Injury scale (ACL-RSI) cutoff score for a return to sports.

**Methods:**

This was a cross-sectional study. Fifty-four patients who had undergone primary reconstruction using hamstring tendon participated in this study. Psychological readiness was measured using the ACL-RSI in patients at 6 months after reconstruction. To identify specific physical functions related to the ACL-RSI score, participants were divided into groups with ACL-RSI scores of ≥ 60 or < 60. Non-paired *t*-tests or the Mann–Whitney test were performed to analyze group differences in objective variables in physical function: (1) knee strength in both legs; (2) leg anterior reach distance on both sides; and (3) single-leg hop (SLH) distances in three directions for both legs.

**Results:**

Significant differences between groups were identified in knee flexion strength (60°/s) for the uninvolved limb, hamstring-to-quadriceps ratio (60°/s) for the uninvolved limb, knee flexion strength (180°/s) for the involved limb, limb symmetry index (LSI) of leg anterior reach distance, the ratio of the distance to the height of the patient and LSI of SLH distances in lateral and medial directions.

**Conclusion:**

This study revealed that at 6 months after reconstruction, increased knee flexion strength (ratio of peak torque measured to body mass of the patient), hamstring-to-quadriceps ratio, leg anterior reach distance LSI, and lateral and medial SLH appear important to exceed the ACL-RSI cutoff for a return to sports. The present results may be useful for planning post-operative rehabilitation for long-term return to sports after reconstruction.

## Background

Many patients who sustain damage to the anterior cruciate ligament (ACL) undergo reconstruction. After ACL reconstruction, these individuals require long-term rehabilitation to improve physical function and return to the sport they participated in before the ACL injury [15, 30]. However, only 44–63% of patients are able to return to their sport [6], and around 17% of elite athletes prove unable to return to their sport [30].

Emotions, confidence, and risk appraisal, in combination representing psychological readiness, contribute to the ability to return to sport after reconstruction [61, 62]. An Anterior Cruciate Ligament–Return to Sport after Injury scale (ACL-RSI) was developed to quantify the state of psychological readiness during recovery after injury and reconstruction [22, 62]. The ACL-RSI score of a patient after reconstruction is related to whether the individual can return to their sport [5, 32, 53, 62, 63]. Lower ACL-RSI scores have been associated with the occurrence of secondary injuries after ACL reconstruction [38, 39]. In recent years, understanding the factors behind post-reconstruction long-term outcomes has increasingly been considered an important part of mitigating the social impacts of ACL damage [16, 54]. Sadeqi et al. [53] performed a multivariate analysis of 681 post-reconstruction patients with outcomes of return or non-return to the same preinjury sport at 2 years after surgery. Only ACL-RSI score at 6 months was included in the final model, and an ACL-RSI score ≥ 60 out of 100 at the 6 months follow-up was the most influential predictor of a return to the preinjury sport as of the 2 years follow-up [53]. In recent years, inclusion of the ACL-RSI score in criteria for the return to sport has been recommended [2–4, 14, 46, 53, 61].

Information about specific factors of physical function that contribute to psychological readiness is needed to plan the rehabilitation for returning to sports. Two previous studies have revealed factors of psychological readiness from objective variables of physical function [1, 64]. The ACL-RSI score of athletes aiming to return to a sport 6 months after reconstruction was affected by symmetry of the lateral single-leg hop (SLH) distance [1]. In that study, knee extension and flexion strength, leg anterior reach distance, and SLH distances in anterior and medial directions were included as independent variables [1]. However, all variables were analyzed as the limb symmetry index (LSI), obtained by dividing the value for the surgical (involved) limb by that for the nonsurgical (uninvolved) limb and multiplying by 100 [1]. In a previous study of patients 12 months after reconstruction, greater LSI of anterior SLH distance had positive effects on the ACL-RSI score [64]. In the previous study, only the LSI of anterior SLH distance was analyzed as an independent variable of physical function [64].

Since the function of the uninvolved limb is reduced after reconstruction, if LSI is used as an index of functional recovery, knee strength and SLH distance of the involved limb may be overestimated [65]. The weight ratio, not the LSI of knee extension strength, has been reported as a factor hindering the return to sports after reconstruction [33]. Therefore, when the ratio of knee peak torque to the body mass of the patient is included among the independent variables, the results for relationships may differ from those of previous studies [1, 64].

The purpose of this study was to identify specific physical functions related to the psychological readiness of patients aiming to return to sports 6 months after reconstruction. Physical functions standardized to body mass, height, and lower limb length were analyzed as independent variables. We hypothesized that the weight ratio of knee strength is a factor related to the ACL-RSI cutoff for a return to sports.

## Methods

### Participants

**Fig. 1:**
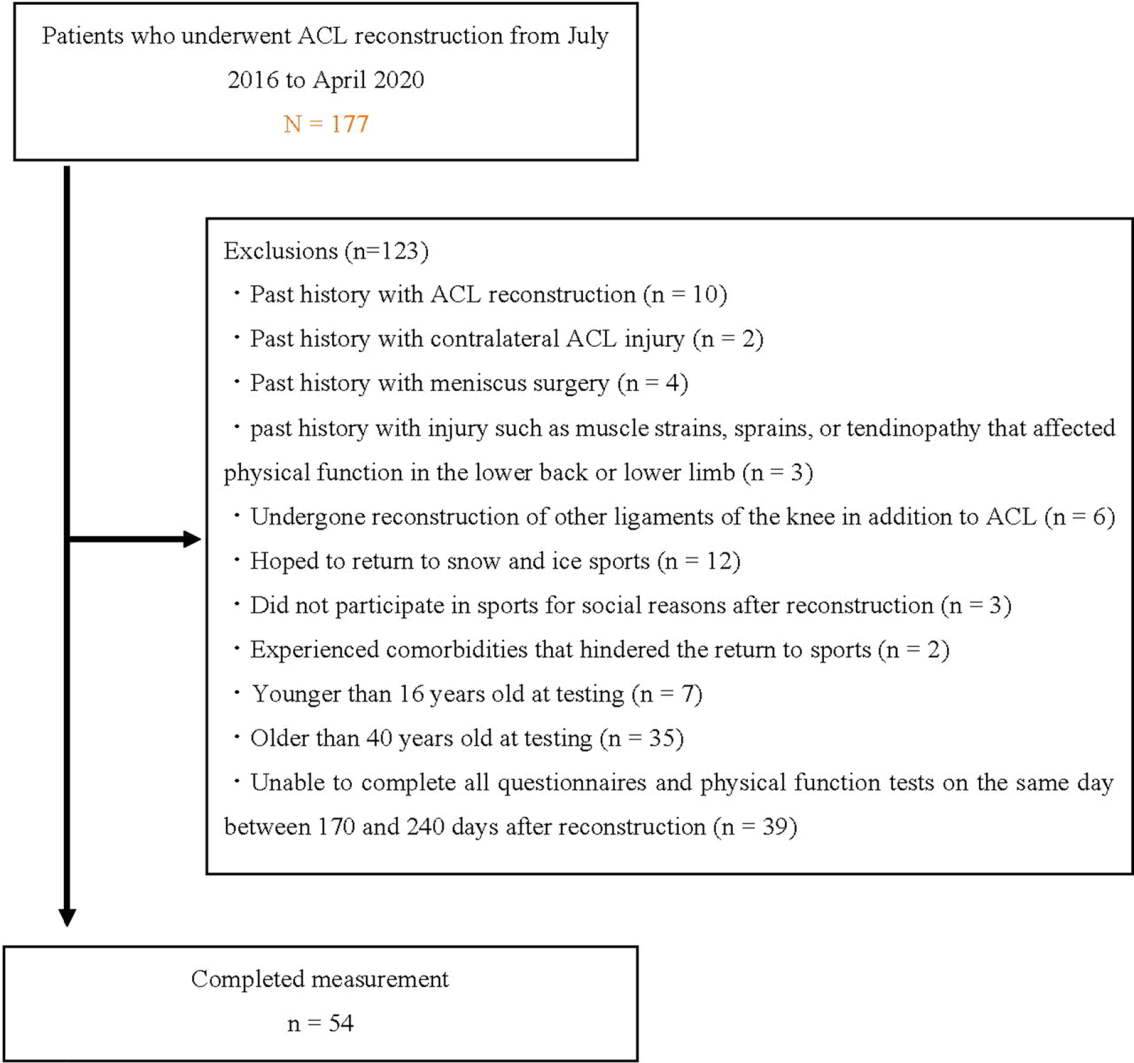
Study flowchart. ACL, anterior cruciate ligament

From July 2016 to the end of April 2020, patients in this cross-sectional study were selected from the list of 177 patients who underwent ACL reconstruction in the Department of Joint Surgery and Sports Medicine at a single center (Fig. 1). The ACL-RSI and physical functions were measured at approximately 6 months after reconstruction. Inclusion criteria were: primary/unilateral anatomical double-bundle reconstruction using either hamstring tendon autograft alone or gracilis tendon harvested in addition to hamstring tendon or patellar tendon autograft; age ≥ 16 years and ≤ 40 years at testing; postoperative rehabilitation with the same protocol used in the sports physical therapy department; and participation in training sessions for the same sport the patient participated in before the ACL injury at approximately 6 months after reconstruction [1].

Patients were excluded if they had: past history with ACL reconstruction; past history with contralateral ACL injury; past history with meniscus surgery; past history with injury such as muscle strains, sprains, or tendinopathy that affected physical function in the lower back or lower limb after reconstruction or in the 6 months before reconstruction; undergone reconstruction of other ligaments of the knee in addition to the ACL; hoped to return to snow and ice sports such as skiing and ice hockey; had not participated in sports for social reasons such as relocating or becoming pregnant after reconstruction; experienced comorbidities that hindered the return to sports; or were unable to complete the ACL-RSI and physical function tests on the same day between 170 and 240 days after reconstruction [1]. Patients wishing to return to snow and ice sports were excluded because these sports involve distinctly different surfaces, shoes, and mechanisms of injury [8, 66].

The institutional review board at our institution approved the study in accordance with the Declaration of Helsinki (approval number: M2019-019). All participants provided written, informed consent prior to enrolment in this trial.

### Postoperative rehabilitation

The postoperative rehabilitation protocol was the same for all patients [26, 42]. However, patients who underwent repair of the middle posterior segment of the meniscus were prohibited from deep squatting until 3 months after surgery [25, 27, 28, 58]. Thirty-two patients underwent repair of the middle posterior segment of the meniscus (lateral meniscus, 26; medial meniscus, 7; both, 1. Patients were permitted to begin isometric quadriceps exercises as tolerated from the day after reconstruction. Using a knee brace (Straighten Position Knee-Joint Immobilizer; ALCARE, Tokyo, Japan) and crutches, partial weight-bearing (20 kg) was permitted on the first day after reconstruction, gradually increasing to full body weight-bearing for each patient. Use of the knee brace and crutches was discontinued at 4 weeks after reconstruction. Range-of-motion exercises from full extension to 120° of flexion were started on the second day after reconstruction. Closed kinetic chain exercises such as weight shifting and squatting were started 1–2 weeks after reconstruction. Patients were instructed to refrain from repeated knee extension training with maximum resistance near the ankle in a sitting position within the range of 10–30° of knee flexion for 3 months after reconstruction [13, 37]. Heel slide exercise was started 3 days after reconstruction. This is an exercise in which the patient bends the knee while sliding the heel on the bed in a long sitting position [55]. Two weeks after reconstruction, curl exercise was started to actively bend the knee in a prone position [7, 10]. Hip lift exercise to raise the buttocks in the crook lying position was started 4 weeks after reconstruction. All exercises were performed while confirming that no pain was present in the area from which the tendon was harvested.

Running exercises were started in athletes who had cleared the criterion of LSI of ≥ 65% of knee isokinetic extension strength as measured by the Biodex Multi-Joint Testing and Rehabilitation System (BDX-4; Biodex Medical Systems, NY, USA) at 3 months after reconstruction. Speed and distance of running were gradually increased for joint effusion and symptoms of each patient. Once 80% of subjective full-speed running ability was achieved, exercises related to the desired sporting activities were initiated with detailed instructions. All exercises were specific to each patient, depending on the type of sport and position played.

Participation in sports exercises with limited contact was allowed from 6 months after reconstruction, as long as the patient showed no problematic symptoms in the joint and displayed sufficient knee isokinetic flexion/extension strength (LSI, > 80%) and showed SLH distance (LSI, > 80%) after the specified training without contact had been completed [26, 42]. Criteria for determining when to return to participation in the actual sport were: ≥ 8 months after reconstruction [1], LSI of flexion/extension strength ≥ 90% [29], LSI of SLH distance ≥ 90% [29], ACL-RSI score ≥ 60 [22, 53], and subjective running ability ≥ 90% [1].

### Measurements

On the same day the ACL-RSI was completed, physical functions were measured. The rest interval between knee strength tests, leg anterior reach test, and SLH tests was 10 min. All physical functions were measured by 5 physiotherapists (J.A., K.H., S.O., T.O., S.M.), each with more than 10 years of clinical experience in rehabilitation for patients after ACL reconstruction.

### Participant characteristics

#### Demographic data

Sex was determined based on medical records. Height and body mass were measured on the same testing day, and body mass index was calculated.

#### Preinjury sports activity level, participation time and type

The level of sports activity before injury was graded using the modified Tegner activity scale [17]. Participants were interviewed regarding the average time (in hours) of participation in sports the week before injury. Types of sports participated in before the injury were classified into four types: collision; contact; limited contact; and non-contact [41].

#### Injury situation, time from injury to reconstruction and reconstruction to testing

Injury situations were elicited from participants, and were classified into three categories: non-contact; indirect contact; and direct contact [59]. The date of injury and date of reconstruction were confirmed both by the participant and from medical records, and the number of days from date of injury to date of reconstruction was calculated. The number of days after reconstruction was the number of days from reconstruction to testing.

#### Autograft

Types of autograft (semitendinosus tendon; semitendinosus plus gracilis tendon; patellar tendon) were confirmed from detailed records made during reconstruction.

#### Meniscal surgery

Meniscus injuries and treatments were confirmed from detailed records of arthroscopic findings during reconstruction. The injured segment (anterior, middle, or posterior), injury type (longitudinal, radial, or horizontal), and treatment method (suture, centralization, or partial meniscectomy) were confirmed. Participants were defined as being treated regardless of the method used.

### Psychological readiness to return to sport

#### ACL-RSI

Patients completed the ACL-RSI, a 12-item scale designed to measure psychological readiness to return to sport after ACL injury or reconstruction [32, 62]. The ACL-RSI includes three domains: emotions; confidence; and risk appraisal. Scores for each domain are summed and averaged for a total score between 0 and 100, with higher scores indicating greater psychological readiness. The scale has been validated and its predictive value has been demonstrated in previous studies [5, 32, 60]. The Japanese version of the ACL-RSI was created and has been confirmed to offer a highly practical questionnaire with good surface validity and internal consistency [22]. In this study, an ACL-RSI cutoff of 60 was used to divide groups [53].

### Outcome scores of physical function

#### Knee strength

The Biodex Multi-Joint Testing and Rehabilitation System (BDX-4; Biodex Medical Systems) was used to evaluate isokinetic strength of the knee in extension/flexion. To minimize compensatory movements during testing, participants were seated and secured with padded straps around the thigh, pelvis, and torso. The femoral condyle of the tested limb was aligned with the rotation axis of the torque meter. Participants performed 3–5 repetitions of submaximal knee extension/flexion to familiarize themselves with the testing motion. To determine the strength of knee extension/flexion, participants performed 5 consecutive concentric contractions of extension/flexion at 60°/s and 180°/s. Peak torque within the 5 trials was extracted and normalized by body mass. Prior to strength measurements, participants were verbally instructed to repeat the cycle of extending and bending the knee as strongly and quickly as possible over the entire range of motion until the end of measurement was declared. No verbal commands were provided during measurements. The uninvolved limb was tested before the involved limb. Five minutes of rest was provided between familiarization and strength tests. The rest interval between strength tests at 60°/s and 180°/s was 5 min. The knee strength test at 60°/s was performed first, followed by the same test at 180°/s. Results are represented as the ratio of the peak torque measured to the body mass of the patient (weight ratio) and LSI. The hamstring-to-quadriceps ratio (HQ ratio) was calculated as the ratio of peak torque of the hamstring to peak torque of the quadriceps. The test–retest reliability of concentric peak torque for the knee using the Biodex System has been reported as high to very high [12, 23, 24, 40, 57].

#### Leg anterior reach distance

Leg anterior reach distance with maximal effort was measured using a Y Balance Test Kit (Functional Movement Systems®, Chatham, VA, USA) [18, 20]. Participants were instructed to perform the leg anterior reach using a combination of verbal cues and demonstration. Participants did not wear shoes during testing, which began on the uninvolved limb. Participants were asked to assume a single-limb stance with the extremity while reaching outside their base of support to push a reach indicator box along the measurement pipe of the kit. Loss of balance resulting in a stepping strategy was recorded as a trial error, indicating the trial should then be repeated. Participants were allowed at least 6 practice trials before recording. Five minutes of rest was provided between practice trial and test. Results are represented as the ratio of the reach distance-to-lower limb length of the patient and LSI.

#### SLH distance

SLH distances in the three directions (anterior, lateral, and medial) were measured in random order according to previous research [1, 19]. Participants stood on one leg and were instructed to hop as far as possible and land on the same leg. The longest distance from 3 trials was recorded for each leg and each direction. The test was considered successful if the landing was stable for 3 s. If the patient landed with early touchdown of the contralateral limb, representing a loss of balance, or took additional hops after landing, the SLH test was repeated. Patients were initially given a verbal description of the test, and were allowed to perform as many practice trials as desired, until they felt confident about the test. Participants were allowed to use the upper limbs as desired during SLH. Three trials were performed for each leg, always starting with the uninvolved limb. Five minutes of rest was provided between familiarization and hop test. The rest interval between anterior, lateral and medial SLH tests was 3 min. For anterior SLH, the distance between the front end of the toe at the starting position and the trailing edge of the heel at the landing position was measured [43]. For lateral SLH, the distance between the lateral side of the foot at the starting position and the medial side of the foot at the landing position was measured. For medial SLH, the distance between the medial side of the foot at the starting position and the lateral side of the foot at the landing position was measured. Results are represented as the ratio of the distance measured to the height of the patient (height ratio) and LSI. Intraclass correlation coefficient (ICC) case 1 was calculated to examine the reproducibility of SLH distances in the three directions for the involved and uninvolved limbs of 10 athletes who met the same inclusion criteria applied in this study. To determine ICCs, SLH distance was measured 3 times in a single day and ICCs of 1–3 measured values were calculated in each direction. As a result, the ICCs of single measurement values of the involved limb and uninvolved limb were within the ranges of 0.91–0.99 and 0.91–0.96, respectively, showing “almost perfect” reproducibility [31]. SLH distances were included because the psychological readiness of athletes aiming to return after reconstruction to limited-contact sports was affected by the SLH variable [1].

### Statistical analysis

Participants were divided using an ACL-RSI cutoff of 60, forming an ACL-RSI ≥ 60 group and an ACL-RSI < 60 group [53]. The following analyses were performed on the characteristic variables and 29 outcome scores for all participants and for each group. The normality of each variable was confirmed using the Shapiro–Wilk test. Normally distributed data for continuous variables are summarized using means and standard deviations. Non-normally distributed data are summarized using medians and interquartile ranges. A 95% confidence interval was calculated for these values. Differences between groups with normally distributed data were analyzed using non-paired *t*-tests. Differences between groups with non-normally distributed data were analyzed using the Mann–Whitney test. Effect sizes by post-test were calculated for all outcome scores. Effect size was judged to be large for ≥ 0.8, medium for ≥ 0.5 but < 0.8, and small for ≥ 0.2 but < 0.5 [11]. The frequency bias of the nominal scale data of characteristics was analyzed using the *χ*^2^ test. All data were analyzed using Statistical Package for the Social Sciences for Windows (version 21.0; IBM Corp., New York, NY, USA). Values of *P* < 0.05 were considered indicative of statistical significance.

## Results

Mean ACL-RSI score for total participants was 64.8. ACL-RSI ≥ 60 included 31 participants and ACL-RSI < 60 included 23 participants. Mean ACL-RSI scores for ACL-RSI ≥ 60 and ACL-RSI < 60 were 77.4 ± 12.1 [73.0–81.9] and 47.8 ± 7.9 [44.4–51.2], respectively, showing a significant difference between groups. Characteristic variables showed no significant differences between groups (Table 1).

**Table 1:** Participant characteristics (N = 54)^a^

	Total (N = 54)	ACL-RSI over 60 points (n = 31)	ACL-RSI under 60 points (n = 23)	*P*
Age, y	20.0 (4.3) [19.9–22.8]	20.0 (8.0) [19.5–23.2]	20.0 (4.0) [19.0–23.9]	0.860
Women: men	33:21	19:12	14:9	0.975
Height, cm	165.8 ± 8.3 [163.5–168.1]	166.1 ± 8.3 [163.1–169.2]	165.4 ± 8.5 [161.7–169.0]	0.744
Body mass, kg	61.0 (16.5) [59.5–66.1]	61.0 (19.0) [58.8–67.4]	59.0 (15.9) [56.9–68.0]	0.733
Body mass index, kg/m^2^	21.9 (3.0) [21.9–23.5]	22.2 (2.8) [21.7–23.7]	21.8 (4.5) [21.3–24.1]	0.740
ACL-RSI score (0–100)	64.8 ± 18.1 [59.9–69.7]	77.4 ± 12.1 [73.0–81.9]	47.8 ± 7.9 [44.4–51.2]	< 0.000*
Preinjury modified Tegner activity scale score	8.0 (2.0) [7.6–8.2]	8.0 (2.0) [7.5–8.4]	8.0 (2.0) [7.5–8.2]	0.898
Preinjury sports participation time, h/wk	6.0 (8.0) [6.6–10.1]	4.0 (8.0) [5.4–9.9]	9.0 (8.0) [6.4–12.1]	0.352
Participating sport type (collision; contact; limited contact; noncontact)	6; 33; 7; 8	3; 21; 4; 3	3; 12; 3; 5	0.237
Injury situations (non-contact; indirect contact; direct contact)	34; 14; 6	19; 7; 5	15; 7; 1	0.269
Time from injury to reconstruction, d	66.0 (76.5) [68.1–150.8]	56.0 (97.0) [57.2–140.9]	67.0 (63.0) [40.0–206.9]	0.576
Meniscus treated:nontreated, n	37:17	22:9	15:8	0.653
Autograft (ST; STG; PT)	44; 4; 6	25; 2; 4	19; 2; 2	0.454
Time from reconstruction to testing, d	186.5 (15.3) [184.0–191.5]	187.0 (15.0) [182.8–191.8]	183.0 (15.0) [181.5–195.2]	0.611

^a^Data are reported as mean ± SD or median (interquartile range) [95% confidence interval] unless otherwise indicated. *ACL-RSI* Anterior cruciate ligament-return to sport after injury scale. *ST* Semitendinosus; *STG* Gracilis tendon in addition to semitendinosus; *PT* Patellar tendon

*Significance at *P* < 0.05

The following variables were significantly larger in ACL-RSI ≥ 60: knee flexion strength (60°/s) for the uninvolved limb, HQ ratio (60°/s) for the uninvolved limb, and knee flexion strength (180°/s) for the involved limb (Table 2). LSI of leg anterior reach distance in ACL-RSI ≥ 60 was significantly larger (Table 2). In the involved limb, height ratio and LSI of lateral and medial SLH distance were significantly larger in ACL-RSI ≥ 60 (Table 2). The ranges of effect size and power of outcome scores, where the difference between groups was significant, were 0.30–0.82 and 0.19–0.83, respectively. Variables for which the effect size was larger than 0.5 were as follows: knee flexion strength (180°/s) of the involved limb, LSI of leg anterior reach distance, lateral SLH distance of the involved limb, LSI of lateral SLH distance, and medial SLH distance of the involved limb.

**Table 2:** Outcome Scores (N = 54)^a^

	Total (N = 54)	ACL-RSI over 60 points (N = 31)	ACL-RSI under 60 points (N = 23)	*P*	Effect size
*Knee extension strength (*60* deg/s)*					
Involved limb, Nm/kg	2.07 ± 0.39 [1.96–2.18]	2.13 ± 0.41 [1.98–2.28]	1.98 ± 0.36 [1.83–2.14]	0.164	0.25
Uninvolved limb, Nm/kg	2.49 ± 0.47 [2.37–2.62]	2.57 ± 0.46 [2.40–2.74]	2.39 ± 0.46 [2.19–2.59]	0.167	0.40
LSI, %	83.74 ± 10.84 [80.78–86.70]	83.53 ± 10.23 [79.77–87.28]	84.03 ± 11.83 [78.91–89.15]	0.867	0.05
*Knee flexion strength (*60* deg/s)*					
Involved limb, Nm/kg	1.08 ± 0.25 [1.01–1.14]	1.12 ± 0.21 [1.05–1.20]	1.01 ± 0.28 [0.89–1.13]	0.098	0.40
Uninvolved limb, Nm/kg	1.29 (0.42) [1.17–1.40]	1.26 (0.34) [1.23–1.45]	1.11 (0.30) [0.98–1.45]	0.026*	−0.30
LSI, %	86.59 ± 16.55 [82.07–91.10]	85.62 ± 14.29 [80.38–90.86]	87.89 ± 19.45 [79.48–96.30]	0.638	0.14
*HQ ratio (*60* deg/s)*					
Involved limb, %	52.26 ± 9.15 [49.76–54.76]	53.35 ± 8.40 [50.27–56.43]	50.79 ± 10.08 [46.43–55.15]	0.315	0.28
Uninvolved limb, %	49.92 (9.50) [48.12–54.70]	52.34 (6.64) [48.92–56.38]	46.46 (10.27) [43.57–55.91]	0.026*	−0.30
*Knee extension strength (*180* deg/s)*					
In involved limb, Nm/kg	1.45 ± 0.26 [1.37–1.52]	1.50 ± 0.24 [1.41–1.59]	1.38 ± 0.27 [1.26–1.49]	0.089	0.40
Uninvolved limb, Nm/kg	1.78 ± 0.31 [1.69–1.86]	1.82 ± 0.29 [1.71–1.92]	1.72 ± 0.33 [1.57–1.87]	0.252	0.33
LSI, %	80.42 (9.66) [79.43–83.94]	80.69 (10.53) [79.55–85.83]	78.66 (10.63) [76.97–83.71]	0.447	−0.10
*Knee flexion strength (*180* deg/s)*					
Involved limb, Nm/kg	0.84 ± 0.22 [0.78–0.90]	0.90 ± 0.18 [0.83–0.96]	0.77 ± 0.24 [0.67–0.88]	0.040*	0.50
Uninvolved limb, Nm/kg	0.97 ± 0.21 [0.91–1.02]	1.00 ± 0.19 [0.94–1.07]	0.91 ± 0.22 [0.82–1.01]	0.116	0.50
LSI, %	87.32 ± 13.48 [83.64–91.00]	89.44 ± 9.43 [85.98–92.90]	84.46 ± 17.37 [76.94–91.97]	0.222	0.37
*HQ ratio (*180* deg/s)*					
Involved limb, %	58.24 ± 10.37 [55.41–61.07]	60.16 ± 9.03 [56.85–63.47]	55.66 ± 11.65 [50.62–60.69]	0.116	0.44
Uninvolved limb, %	54.50 ± 7.62 [52.42–56.58]	55.57 ± 7.98 [52.65–58.50]	53.04 ± 7.02 [50.01–56.08]	0.232	0.34
*Leg anterior reach distance*					
Involved limb, % lower limb length	69.8 ± 6.5 [68.0–71.6]	70.3 ± 6.7 [67.8–72.8]	69.1 ± 6.3 [66.4–71.9]	0.524	0.18
Uninvolved limb, % lower limb length	72.0 ± 6.4 [70.2–73.8]	71.5 ± 7.1 [68.9–74.1]	72.7 ± 5.5 [70.4–75.1]	0.488	0.19
LSI, %	97.0 ± 4.7 [95.7–98.3]	98.5 ± 3.8 [97.1–99.8]	95.1 ± 5.3 [92.8–97.4]	0.008*	0.76
*Anterior SLH distance*					
Involved limb, % height	62.3 ± 15.1 [58.2–66.4]	65.4 ± 14.7 [60.1–70.8]	58.1 ± 14.9 [51.7–64.5]	0.077	0.49
Uninvolved limb, % height	71.5 ± 14.5 [67.7–75.7]	73.0 ± 16.2 [67.1–79.0]	70.0 ± 12.0 [64.7–75.2]	0.449	0.21
LSI, %	89.6 (16.5) [83.6–90.8]	90.4 (13.1) [86.7–93.9]	86.5 (25.5) [76.1–89.9]	0.139	−0.20
*Lateral SLH distance*					
Involved limb, % height	47.6 ± 12.6 [44.1–51.0]	50.6 ± 12.1 [46.2–55.1]	43.4 ± 12.4 [38.1–48.8]	0.036*	0.59
Uninvolved limb, % height	56.1 ± 12.0 [52.8–59.3]	57.6 ± 13.1 [52.8–62.4]	54.0 ± 10.3 [49.5–58.4]	0.279	0.30
LSI, %	84.5 ± 13.3 [80.9–88.1]	88.3 ± 11.4 [84.1–92.5]	79.3 ± 14.2 [73.2–85.5]	0.013*	0.71
*Medial SLH distance*					
Involved limb, % height	52.1 ± 13.7 [48.4–55.9]	56.4 ± 13.8 [51.3–61.4]	46.4 ± 11.7 [41.4–51.5]	0.007*	0.77
Uninvolved limb, % height	58.4 ± 12.4 [55.0–61.8]	60.6 ± 13.6 [55.6–65.6]	55.6 ± 10.2 [51.2–60.0]	0.146	0.41
LSI, %	89.5 (16.1) [85.1–92.9]	93.2 (13.5) [89.0–97.7]	82.9 (19.1) [76.5–89.8]	0.018*	−0.32
Subjective running ability, %	80.0 (30.0) [79.5–87.5]	95.0 (20.0) [85.3–93.9]	80 (10.0) [69.1–81.4]	0.001*	−0.47
Tampa scale for kinesiophobia	35.6 ± 6.5 [33.9–37.4]	33.5 ± 6.2 [31.2–35.7]	38.5 ± 5.9 [36.0–41.1]	0.004*	0.82

^a^Data are reported as mean ± SD or median (interquartile range) [95% confidence interval]. *LSI* Limb symmetry index; *SLH* Single-leg hop

*Significance at *P* < 0.05

## Discussion

We hypothesized that the weight ratio of knee strength is a factor related to the ACL-RSI cutoff for a return to sports. Differences between groups were seen in the weight ratio of knee flexion strength and the HQ ratio. The present results supported our hypothesis. Knee flexion strength at different angular velocities in uninvolved limbs as well as involved limbs should be measured and postoperative rehabilitation to increase these strengths should be planned. In addition, the HQ ratio of uninvolved limb as well as involved limb should be calculated, with plans to strengthen the force of knee flexion against knee extension.

The weight ratio of knee flexion strength and HQ ratio is the factor related to the ACL-RSI cutoff for a return to sports. In this study, the following muscle strength variables were significantly larger in ACL-RSI ≥ 60 than in ACL-RSI < 60 at 6 months after reconstruction: knee flexion strength (180°/s) of the involved limb, knee flexion strength (60°/s) of the uninvolved limb, and HQ ratio (60°/s) of the uninvolved limb. Performances such as sprint times and jump distances are affected by hamstring function [21, 51]. During jump landings and cutting, the anterior shear and rotational forces of the tibia are controlled by hamstring function [35, 36]. Excessive strain in the graft is suppressed by the hamstring [35]. For these reasons, knee flexion strength and HQ ratio were significantly higher in the group with better psychological readiness.

Aizawa et al. reported no significant association between ACL-RSI score and knee flexion/extension strength LSI in patients 6 months after reconstruction [1]. Müller et al. reported no significant association between ACL-RSI score and knee flexion/extension force LSI or HQ ratio LSI in patients at 6 months after reconstruction [45]. O’Connor et al. [47] analyzed the relationship between ACL-RSI score and the weight ratio of knee extension/flexion strength of the involved limbs in patients 9 months after reconstruction. In that study, only the weight ratio of knee flexion strength for the involved limb correlated significantly with ACL-RSI score, and all correlation coefficients including this variable were less than 0.2, indicating a weak association [47]. In that previous study, the weight ratio of knee extension/flexion strength and LSI were compared between an ACL-RSI ≥ 90 group and an ACL-RSI < 75 group, and the only significant difference seen between groups was for the weight ratio of knee flexion strength [47]. However, the effect size was concluded to be 0.15, with no meaningful difference found [47]. In the present study, the flexion strength variable, rather than knee extension, showed a significant difference between groups, with effect sizes in the 0.30–0.50 range. These results appear to support the findings of O’Connor et al. Previous studies did not analyze uninvolved limb knee HQ ratios or flexion weight ratios.

Regarding leg anterior reach distance, LSI was shown to be a factor related to the ACL-RSI cutoff for a return to sports. In this study, the LSI of leg anterior reach distance was significantly larger in the ACL-RSI ≥ 60 group than in the ACL-RSI < 60 group at 6 months. In post-reconstruction patients, lower limb strength and neuromuscular control are required for the task of reaching one lower limb forward while standing on the other leg [20, 44, 50]. In a healthy netball player, leg anterior reach distance and knee rotation moment during one-leg landing show a negative correlation [9]. Leg anterior reach distance asymmetry is associated with the timing of return to sports after reconstruction [18]. For these reasons, the LSI of leg anterior reach distance was significantly larger in the group with better psychological readiness.

Some individuals who meet the criteria for returning to sports after reconstruction have shown a significantly shorter leg anterior reach distance on the operated lower limb than on the non-operated side [49]. Leg anterior reach distance LSI was significantly associated with the Knee injury and Osteoarthritis Outcome Score (KOOS)-Symptom (r = 0.30) and KOOS-Sport (r = 0.30) in patients 6 months after reconstruction [52]. The present results partially supported the findings from those previous studies. However, previous studies have not analyzed the relationship between ACL-RSI score and leg anterior reach distance, and the present study thus provides new information on this relationship.

For SLH, lateral and medial SLH variables were shown to be factors related to the ACL-RSI cutoff for a return to sports. In this study, the following SLH variables were significantly higher in the group with ACL-RSI ≥ 60 than in the group with ACL-RSI < 60 at 6 months: lateral SLH distance of the involved limb, LSI of lateral SLH distance, medial SLH distance of the involved limb, LSI of medial SLH distance. Insufficient jump landing and balance are subjective factors in injury-related fear [48]. Kinetics such as knee valgus associated with re-injury show a worse pattern in lateral SLH than in anterior SLH [56]. For these reasons, lateral SLH distance and LSI may have been significantly smaller in the group with poorer psychological readiness.

Aizawa et al. identified lateral SLH distance LSI as a factor in ACL-RSI score among patients 6 months after reconstruction by simple regression analysis (β coefficient = 0.58, P = 0.031) [1]. The present study supported some of the findings from that study. Müller et al. showed that anterior SLH distance LSI was weakly associated with ACL-RSI score in patients 6 months after reconstruction (Pearson’s r = 0.36, *P* = 0.023) [45]. Webster et al. revealed by simple regression analysis that the LSI of anterior SLH distance is a factor in ACL-RSI score for patients at 12 months after reconstruction (β coefficient = 0.50, *P* = 0.001) [64]. In the present study, no difference was observed between groups in the LSI of anterior SLH distance, and our results did not support the findings of previous studies. In this study and past investigations, age and postoperative period of subjects differed, which may be one reason for differences in the relationship between the LSI of anterior SLH distance and ACL-RSI score.

The present study showed some limitations that merit consideration. First, the causal associations between physical functions and ACL-RSI cutoff are unclear, given the cross-sectional nature of the study. Second, in this study, an ACL-RSI score of 60 at 6 months after reconstruction, which is related to the return to sports by 2 years after reconstruction, was used as the cutoff [53]. Müller et al. reported a cutoff score for ACL-RSI of 51.3 associated with a return to preinjury level sports in patients at 6 months after reconstruction [45]. The ACL-RSI cutoff at 6 months after surgery will differ depending on when the outcome of returning to sports after reconstruction is judged. Third, limits exist to generalizing the results of this study to patients with significantly different characteristics, such as age and sex, surgical procedures including meniscus treatment and autograft type, different sports, and postoperative days before returning to the sport [32, 34]. Fourth, in this study, multiple variables differed significantly between groups. However, for variables with low effect size and power, type 2 errors must be considered.

## Conclusion

This study revealed that at 6 months after ACL reconstruction, increasing knee flexion strength-weight ratio, HQ ratio, leg anterior reach distance LSI, and lateral SLH appear important to exceed the ACL-RSI cutoff for a return to sports. The present results may be useful for planning post-operative rehabilitation for long-term return to sports after reconstruction.

## Data Availability

The datasets used and/or analyzed during the current study are available from the corresponding author on reasonable request.

## Abbreviations

ACL: Anterior cruciate ligament
ACL-RSI: Anterior cruciate ligament–return to sport after injury scale
SLH: Single-leg hop
LSI: Limb symmetry index
Weight ratio: Ratio of peak torque measured to body mass of the patient
HQ ratio: Hamstring-to-quadriceps ratio
Height ratio: Ratio of the distance measured to the height of the patient
ICC: Intraclass correlation coefficient
